# Comparing 2-Hz and 100-Hz Electroacupuncture for Postoperative Musculoskeletal Pain: Protocol for a 2 × 2 Prospective Randomized Crossover Trial

**DOI:** 10.2196/91706

**Published:** 2026-06-16

**Authors:** Jeong Hwan Park, Hyeong Joon Jun, Young-Eun Kim, Sanghun Lee, Daehyeok Kim, Sunmi Choi

**Affiliations:** 1 Korea Institute of Oriental Medicine Daejeon Republic of Korea

**Keywords:** electroacupuncture, chronic postsurgical pain, pulse rate variability, radial pulse tonometry, acupoint, crossover trial

## Abstract

**Background:**

Postoperative pain after musculoskeletal surgery impedes early rehabilitation and reduces quality of life. Electroacupuncture is used as an adjunct for analgesia, but the optimal stimulation frequency and short-term response trajectory have not been established.

**Objective:**

The aim of this study is to compare frequency-dependent relative analgesic responses to bilateral ST36 to SP9 electroacupuncture at 2 Hz and 100 Hz and generate effect size and variability estimates to inform future definitive trials.

**Methods:**

This single-center, frequency-masked, active-comparator, 2 × 2 prospective randomized crossover trial will enroll 30 adults (aged 19-80 years) experiencing pain (numerical rating scale [NRS] score≥4) after musculoskeletal surgery. Participants will undergo two 15-minute electroacupuncture sessions at bilateral ST36 to SP9 (2 Hz and 100 Hz) separated by a washout period of at least 7 days. Participants will not be informed of the assigned stimulation frequency or treatment sequence.

**Results:**

Participant recruitment began in September 2025, and data collection is expected to be completed by August 2026. Data analysis will begin after database finalization in late 2026, with primary findings submitted for publication in April 2027. The primary outcomes are baseline-adjusted changes in pain NRS scores at 15 minutes and 2 hours after each intervention. Secondary outcomes include pain NRS scores immediately after and at 1, 4, and 24 hours after electroacupuncture; pain location and quality; photoplethysmography-derived pulse rate variability, used as a surrogate for heart rate variability; radial pulse tonometry parameters; and wrist-worn activity metrics.

**Conclusions:**

This protocol describes an active-comparator crossover trial designed to estimate relative immediate and short-term analgesic responses to 2-Hz vs 100-Hz electroacupuncture and associated physiological measures in postoperative musculoskeletal pain. Because the trial does not include a sham, usual care, or no-additional-electroacupuncture control arm, it will not determine the absolute efficacy of electroacupuncture or separate specific effects from nonspecific contextual or placebo effects. Results from this trial will help optimize stimulation parameters and outcome assessments for larger, definitive efficacy trials.

**Trial Registration:**

Clinical Research Information Service KCT0011009; https://tinyurl.com/54phyep7

**International Registered Report Identifier (IRRID):**

DERR1-10.2196/91706

## Introduction

Musculoskeletal procedures such as total knee arthroplasty, spinal surgery, and total hip arthroplasty are becoming increasingly common with population aging. These procedures improve functional outcomes and reduce pain. However, postoperative pain often persists, and this can delay early mobilization and rehabilitation. Some patients develop chronic postsurgical pain [1-3]. Neuropathic components frequently coexist with and complicate pain control after spinal surgery [4]. Pharmacological strategies alone are insufficient for many patients, and current guidelines emphasize multimodal, multidisciplinary pain management [5-7]. Inadequate acute pain control after total knee arthroplasty can lead to chronic pain and long-term decrements in function and quality of life [8-10].

Electroacupuncture is a minimally invasive adjunctive analgesic option. Clinical trials and systematic reviews in arthroplasty populations suggest benefits in pain reduction, opioid sparing, and functional recovery [11,12]. Compared with other neuromodulation techniques such as transcutaneous or peripheral nerve stimulation, electroacupuncture is less invasive and easier to deploy at the bedside [13,14].

The evidence base for electroacupuncture after arthroplasty has expanded, but many crossover or repeated-exposure trials on postoperative pain have not clearly specified a washout period or formally assessed carryover effects [15,16]. The analgesic effect of a single acupuncture session may wane over tens of minutes to several days (approximately 30-72 hours) depending on the condition [17,18], whereas more invasive neuromodulation such as spinal cord stimulation can show residual effects for up to 5 days [19]. On the basis of this, our crossover trial adopts a conservative washout period of at least 7 days to minimize carryover.

The analgesic effects of electroacupuncture are frequency dependent. Low-frequency stimulation (2 Hz) preferentially elicits β-endorphin and enkephalin release, whereas high-frequency stimulation (100 Hz) engages dynorphin-mediated pathways. These findings suggest that the peptide systems are partially distinct [20]. Responsiveness varies across individuals, and intrinsic factors may contribute to this heterogeneity [21]. Procedure- and patient-specific factors also influence postoperative pain intensity [9,22]. Therefore, a 2 × 2 crossover design with intraindividual comparisons is well suited to compare the effects of 2-Hz and 100-Hz stimulation and control for interindividual variability.

The primary hypothesis of this study is that 2-Hz and 100-Hz electroacupuncture will produce different short-term analgesic responses, measured as baseline-adjusted changes in pain numerical rating scale (NRS) scores at 15 minutes and 2 hours after stimulation. This frequency-masked, active-comparator, 2 × 2 randomized crossover study aims to compare relative responses to bilateral ST36 to SP9 electroacupuncture at 2 Hz and 100 Hz.

## Methods

### Study Registration

The study has been registered with the Clinical Research Information Service under registration number KCT0011009.

### Patient and Public Involvement

Patients and/or the public were not involved in the design, conduct, reporting, or dissemination plans of this research.

### Study Design

**Figure 1:**
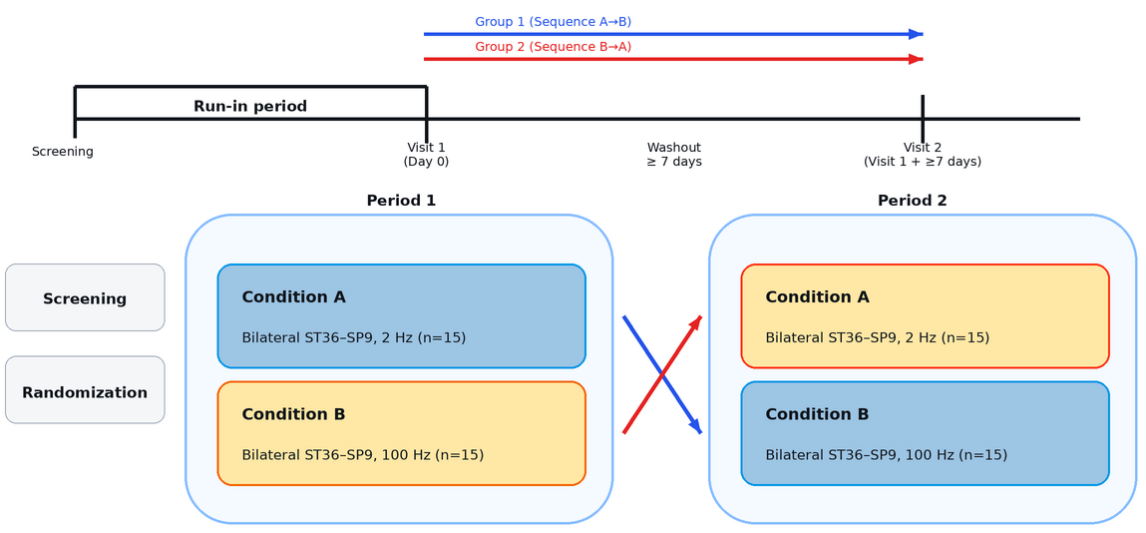
Flowchart of the study design.

This will be a single-center, frequency-masked, active-comparator, 2 × 2 prospective randomized crossover trial. Because no sham, usual care, or no-additional-electroacupuncture arm will be included, electroacupuncture administration itself is open label; however, participants will not be informed of the stimulation frequency applied at each visit or of the treatment sequence. Each participant will present for 2 treatment visits (visits 1 and 2) separated by a washout period of at least 7 days. This duration was selected as a conservative measure to minimize potential carryover of analgesic effects. Participants will be randomized (1:1) to 1 of 2 sequence groups: group 1 will receive mode A (2 Hz) followed by mode B (100 Hz), and group 2 will receive the reverse sequence (mode B followed by mode A). This design enables within-participant comparisons of immediate and short-term responses to the 2 frequencies while reducing interindividual variability (Figure 1). This protocol was prepared primarily with reference to the SPIRIT (Standard Protocol Items: Recommendations for Interventional Trials) 2025 guidelines for randomized controlled trial protocols [23]. Reporting elements specific to the crossover design were additionally supplemented with reference to the CONSORT (Consolidated Standards of Reporting Trials) extension for randomized crossover trials [24].

### Eligibility Criteria

The inclusion criteria will be as follows: (1) age of 19 to 80 years, (2) postoperative musculoskeletal pain with an NRS score of 4 or higher at enrollment, and (3) ability to complete questionnaires and provide written informed consent. The date and type of the index surgery, postoperative day at screening, and postoperative day at each treatment visit will be recorded.

The exclusion criteria will be as follows: (1) pain not attributable to the index surgery or underlying musculoskeletal condition, (2) pregnancy or breastfeeding, (3) having undergone acupuncture or electroacupuncture within 7 days before screening, (4) participation in another clinical trial within 1 month or scheduled surgery within 2 weeks, (5) severe phobia of acupuncture or electrical stimulation or known adverse reactions, (6) contraindications at the needling sites (eg, fresh incision or suture line, infection, severe edema due to deep vein thrombosis, or malignancy), (7) uncontrolled major neuropsychiatric disorders (eg, major depression, schizophrenia, and bipolar disorder), and (8) any condition that the investigators consider to preclude safe participation.

### Interventions

Electroacupuncture will be administered by a licensed doctor of Korean medicine with more than 5 years of clinical acupuncture experience after completion of study-specific protocol training. Electroacupuncture will be administered at bilateral ST36 (Zusanli) and SP9 (Yinlingquan) using sterile, disposable needles (0.20 × 30 mm) connected to a medical electroacupuncture stimulator. Needles will be inserted to a depth of 15 to 25 mm for ST36 and 10 to 20 mm for SP9, with minor adjustments permitted based on individual anatomy and clinical safety. If insertion depth differs from the predefined range for safety or clinical reasons, the reason will be recorded in the electronic case report form (eCRF). A single 15-minute continuous stimulation at the assigned frequency will be administered at each visit. The current intensity will be gradually increased to the lowest clearly perceptible level that remains comfortable and tolerable for each participant to standardize sensory engagement while ensuring tolerability. Achievement of the qi sensation will not be required for treatment completion. Needles will be removed immediately after completion of the 15-minute electrical stimulation. If a participant reports severe discomfort, pain, dizziness, vasovagal symptoms, bleeding, skin abnormalities, or any other adverse event (AE) or requests discontinuation, electrical stimulation will be stopped immediately, and the needles will be removed. Electroacupuncture will be administered in 2 modes: mode A (2-Hz electroacupuncture at bilateral ST36-SP9) and mode B (100-Hz electroacupuncture at bilateral ST36-SP9).

### Physiological Measurements and Activity Monitoring

Pulse rate variability (PRV) will be measured using the ubpulse T1 device (LAXTHA Inc), which is based on photoplethysmography. The device analyzes pulse-to-pulse intervals derived from photoplethysmography pulse waves; therefore, the outcome will be described as photoplethysmography-derived PRV, used as a surrogate for heart rate variability. A photoplethysmography clip sensor will be attached to the tip of the left index finger. For PRV measurement, after the photoplethysmography clip sensor is attached, an additional 2- to 3-minute stabilization period will be allowed before the 1-minute recording. After completion of the measurement, the sensor will be removed according to the software instructions. The collected signals will be analyzed using the manufacturer-provided software according to predefined artifact handling and analysis procedures.

Radial pulse tonometry will be standardized at the right radial artery. If movement occurs during radial pulse tonometry and data error is suspected, the measurement will be repeated. The activity device will be worn on the left wrist whenever feasible. The activity device will be attached immediately following the intervention and removed upon completion of the 2-hour in-clinic observation period.

Electroacupuncture, PRV, and radial pulse tonometry will be performed in the supine position whenever feasible. If the supine position is clinically contraindicated or intolerable for the participant, the sitting position will be permitted and documented in the eCRF. For each participant, the same position will be maintained for baseline and postintervention measurements whenever feasible. During PRV and radial pulse tonometry measurements, participants will be instructed to avoid talking, unnecessary movement, and posture changes. Wrist-worn activity monitoring is intended to record actual activity during the 2-hour in-clinic observation period; physiological measurements will not be performed during active movement. Participants will remain in a designated observation area; however, minimal movement will be permitted when required for safety or clinically appropriate reasons.

### Schedule of Enrollment, Interventions, and Assessments

The participants will undergo screening and baseline assessments before randomization and electroacupuncture administration at 2 Hz or 100 Hz during visit 1. Pain NRS scores will be recorded immediately before the intervention and immediately after and at 15 minutes, 1 hour, 2 hours, 4 hours, and 24 hours after electroacupuncture. Photoplethysmography-derived PRV and radial pulse tonometry data will be recorded at baseline and after 2 hours under standardized resting conditions. The same schedule will be followed for visit 2 after the 7-day or longer washout period.

### Outcome Measures

The primary outcomes are baseline-adjusted changes in pain intensity (11-point NRS; 0-10) at 15 minutes and 2 hours after each intervention. Change scores will be calculated as the postintervention NRS score at each specified time point minus the visit-specific preintervention baseline NRS score. The 15-minute time point was selected to evaluate immediate analgesic response after the 15-minute electroacupuncture stimulation, and the 2-hour time point was selected to evaluate whether the short-term analgesic response is maintained during the standardized in-clinic observation period.

Secondary outcomes will include pain NRS scores immediately after and at 1, 4, and 24 hours after electroacupuncture; pain location and quality; Photoplethysmography-derived PRV indexes (eg, total power, low frequency, high frequency, very low frequency, and low frequency–to–high frequency ratio); radial pulse tonometry parameters (eg, stroke volume, stroke volume index, cardiac output, systemic vascular resistance index, and waveform features); and physical activity metrics (steps, distance, and energy expenditure) from a wrist-worn device during the 2-hour in-clinic observation period.

### Randomization, Allocation, and Blinding

Participants will be randomized in a 1:1 ratio to 1 of 2 treatment sequences (AB or BA) using block randomization with permuted blocks of varying sizes based on a computer-generated allocation list created by an independent statistician. To ensure allocation concealment, a study coordinator not involved in enrollment will prepare opaque, sequentially numbered, sealed envelopes containing the sequence codes and store them securely until assignment. Site investigators will enroll eligible participants. At each visit, the treating practitioner will confirm the assigned intervention immediately before treatment, and the contents of the allocation envelope will not be disclosed to participants or outcome assessors. The assigned randomization number will be recorded in the eCRF, and opened envelopes will be retained separately to maintain an audit trail. Participants will be informed that 2 electroacupuncture stimulation modes are being compared but will not be told whether 2 Hz or 100 Hz are applied at each visit. The treating practitioner cannot be blinded because the electroacupuncture device must be set to the assigned frequency. Outcome assessors collecting pain NRS scores and personnel responsible for data entry and statistical analysis will remain blinded to frequency allocation whenever feasible. Although participants will not be informed of the stimulation frequency assigned at each visit, perceived sensory characteristics, such as rhythm or pulsation, may differ between 2-Hz and 100-Hz stimulation. Therefore, some participants may infer the stimulation mode, and participant masking to stimulation frequency may be incomplete.

### Sample Size

A total of 26 evaluable participants will provide approximately 80% power (2-sided α=.05) to detect a clinically meaningful 1.0-point difference in pain NRS change scores between stimulation frequencies assuming an SD of 2.0 and a within-subject correlation of 0.6 [25-28]. To account for potential sequence imbalances and an anticipated 10% dropout rate, 30 participants will be enrolled. This sample size is planned for within-participant comparison of stimulation frequencies and is not intended to establish the effectiveness of electroacupuncture compared with no electroacupuncture.

### Recruitment Strategy

To recruit participants, promotional posters or informational materials will be displayed within the conducting institution. If participant enrollment is delayed during the clinical trial, local advertisements, such as in subways, buses, and apartment bulletin boards, may be implemented, and online advertisements may also be used.

### Statistical Analysis

The primary analysis will be conducted according to the intention-to-treat principle. A per-protocol analysis will be performed as a sensitivity analysis. Linear mixed models with the participant as a random effect will be used to determine the effects of treatment (2 Hz vs 100 Hz), time, period, and sequence on the changes in NRS scores from baseline. To account for baseline differences, the visit-specific baseline NRS score will be included as a covariate in the linear mixed models, and days since surgery will be treated as an additional covariate or sensitivity analysis variable. Age will be summarized as a baseline characteristic, considered as a covariate in exploratory sensitivity analyses if performed, and interpreted as hypothesis generating because the study is not powered for subgroup comparisons. Intravisit pretest-posttest comparisons may be performed using paired tests for descriptive support. Missing data will be handled using mixed models without imputation. However, they will be imputed using multiple imputation for sensitivity analyses. Statistical analyses will be performed using R (version 4.5.0 or later; R Foundation for Statistical Computing).

### Data Collection and Management

Data will be recorded in a validated eCRF. The date and type of index surgery, postoperative day at screening, postoperative day at each intervention visit, visit-specific baseline NRS score, concomitant analgesic use, and any deviations from standardized measurement procedures will be recorded. Quality control, audit trails, query handling, and backups will be conducted in accordance with institutional standard operating procedures. Access will be role based, and identifiable information will be stored separately from the analytical datasets.

### AE Monitoring and Management

AEs, defined as any harmful or unintended symptoms or abnormalities, will be recorded from informed consent to the end of follow-up. For each AE, the event type, onset date, severity, and outcome or resolution will be documented, and any serious AEs will be reported to the institutional review board (IRB) and relevant authorities according to institutional policy. All AEs will be managed using standard medical procedures and routine clinical protocols to ensure participant safety throughout the study.

### Informed Consent and Consent Acquisition

The researcher will explain the purpose, methods, risks, and participant protections of the study before any clinical procedures. The participants will provide voluntary consent to take part and sign an informed consent form. They will be provided with a copy of the form, and the institution will retain the original. Separate consent will be obtained for data sharing with third parties, secondary research use, and the collection and use of personal data.

### Ethical Considerations

This study protocol (ICT_25_3-2; version 1.1) was approved by the IRB of Catholic Kwandong University International St. Mary’s Hospital on August 27, 2025 (approval 25-IRB-050-1). Any modifications to the protocol will require a formal amendment, which must be reviewed and approved by the IRB before implementation.

### Data Monitoring

Designated monitors will conduct regular on-site visits (once for every 5 participants enrolled) and maintain telephone contact with the study site as needed. During visits, monitors will primarily review source documents, study intervention and device accountability logs, and essential research files while also assessing overall trial progress and promptly discussing any issues with the investigator if problems arise. Access to medical records will be limited to what is necessary, and confidentiality will be protected via password-restricted eCRFs and worksheets. Given the low anticipated risk of the intervention and the absence of a planned interim analysis, a data monitoring committee will not be convened.

## Results

The study was funded in January 2025. Participant recruitment began in September 2025. As of June 8, 2026, 30 participants had been recruited. Data collection is expected to be completed by August 2026, and formal outcome analyses are planned to begin after completion of data collection and database finalization in late 2026. A manuscript reporting the primary findings is expected to be submitted for publication in April 2027.

## Discussion

This randomized active-comparator crossover trial is expected to clarify whether short-term pain reduction differs between 2-Hz and 100-Hz electroacupuncture in patients with postoperative musculoskeletal pain. These results will directly inform the optimal frequency selection and outcome assessment timing for future definitive efficacy trials. The paired physiological measurements are expected to provide preliminary information on whether short-term analgesic responses are accompanied by changes in photoplethysmography-derived PRV and radial pulse tonometry parameters.

The rationale for comparing 2-Hz and 100-Hz electroacupuncture is based on frequency-dependent analgesic mechanisms. Low-frequency electroacupuncture (2 Hz) is expected to preferentially activate β-endorphin and enkephalin pathways with descending serotonergic and noradrenergic inhibition, whereas high-frequency electroacupuncture (100 Hz) is expected to engage dynorphin-mediated spinal antinociception [20]. We hypothesize that these distinct pathways will contribute to observable differences in both subjective pain relief and objective physiological measures. This protocol extends prior postoperative electroacupuncture research by using an intraindividual crossover design, a conservative washout period, and concurrent patient-reported and physiological measures to characterize short-term frequency-dependent responses.

The strengths of this trial include its randomized crossover design, within-participant comparison of 2 active electroacupuncture frequencies, standardized bilateral ST36 to SP9 stimulation, prespecified short-term outcome time points, and concurrent physiological measurements. The frequency-masked procedure, separation of the outcome assessor from the treating practitioner whenever feasible, and blinded statistical analysis are intended to reduce bias related to subjective pain assessment.

Certain limitations should be considered. First, as an active-comparator trial lacking a sham or inactive control, this study is designed to compare frequency-dependent responses rather than evaluate the absolute efficacy of electroacupuncture or control for placebo effects. Second, although participants will not be informed of the assigned stimulation frequency, masking may be incomplete because sensory perceptions can differ between 2-Hz and 100-Hz stimulation. Third, this is a single-center study with a modest sample size, which limits generalizability and precludes meaningful subgroup analyses. Fourth, postoperative pain may change with the recovery phase; therefore, days since surgery will be recorded and considered in the analysis. Finally, PRV derived from photoplethysmography will be used as a surrogate for electrocardiogram-derived heart rate variability, and findings from PRV should be interpreted accordingly.

The findings will be used to estimate effect size and variability, refine outcome timing, and inform sample size calculations for a future definitive trial. Future studies should consider including sham, usual care, or no-additional-electroacupuncture control groups; assessor blinding where feasible; multicenter recruitment; and stratification by surgical procedure, pain phenotype, analgesic use, and postoperative recovery phase. The results of this trial will be disseminated through peer-reviewed publications and conference presentations.

## Data Availability

The data used for analysis in this study are available upon request from the corresponding author. These data are not publicly available because of privacy concerns.

## Abbreviations

AE: adverse event
CONSORT: Consolidated Standards of Reporting Trials
eCRF: electronic case report form
IRB: institutional review board
NRS: numerical rating scale
PRV: pulse rate variability
SPIRIT: Standard Protocol Items: Recommendations for Interventional Trials
